# Training health professionals in infodemic management to mitigate the harm caused by infodemics

**DOI:** 10.1093/eurpub/ckac129.324

**Published:** 2022-10-25

**Authors:** TD Purnat, C Bertrand-Ferrandis, B Yau, A Ishizumi, B White, S Briand, T Ngueyn

**Affiliations:** High Impact Events Preparedness, WHO, Geneva, Switzerland

## Abstract

**Background:**

Due to their multifaceted impacts on health and society, understanding and controlling infodemics to support uptake of vaccines, public health and social measures, treatments, and health behaviours is rapidly becoming a priority for many health authorities. WHO has developed a comprehensive training programme to support health professionals in the new field of expertise of managing infodemics.

**Objectives:**

The WHO infodemic management multiformat and transdisciplinary training program builds the skills and knowledge needed to prepare for and respond to infodemics. The trainings are built on WHO competency framework for building an infodemic response workforce. The methodology used relies on human-centred and emotional design evidence and practice and uses evaluation for continuous learning design improvement.

**Results:**

Since November 2020, three WHO global trainings organized online with US Centers for Disease Control and Prevention, UNICEF and other partners, including four-week-long simulation exercises, creating a network of 772 infodemic managers in 133 countries. A “train-the-trainers” companion package was prepared and by April 2022 delivered in Iran and Malaysia. Deep dive training modules on specialist infodemic management practice topics have been prepared for use at country level. In addition, a comprehensive set of self-paced free online courses enhances infodemic literacy and resilience to misinformation. Between December 2021 and April 2022, the OpenWHO Infodemic Management 101 course reached over 17 000 enrolments. The training programme will be updated based on evaluations, the feedback from field responders and the updated to the WHO competency framework for infodemic management workforce.

**Conclusions:**

The WHO multiformat blended training program allows an efficient and rapid dissemination of infodemic management skills and knowledge.

**Key messages:**

• A global network of trained infodemic managers is ready to support national preparedness and response planning.

• Skills and knowledge in infodemic management are accessible to all thanks to free online courses.

